# Messenger RNA and MicroRNA transcriptomic signatures of cardiometabolic risk factors

**DOI:** 10.1186/s12864-017-3533-9

**Published:** 2017-02-08

**Authors:** David D. McManus, Jian Rong, Tianxiao Huan, Sean Lacey, Kahraman Tanriverdi, Peter J. Munson, Martin G. Larson, Roby Joehanes, Venkatesh Murthy, Ravi Shah, Jane E. Freedman, Daniel Levy

**Affiliations:** 1 Cardiology Division, Department of Medicine, University of Massachusetts Medical School, Worcester, MA USA; 2 National Heart Lung and Blood Institute’s and Boston University’s Framingham Heart Study, Framingham, MA USA; 3 Epidemiology Division, Department of Quantitative Health Sciences, University of Massachusetts Medical School, Worcester, MA USA; 4 Department of Biostatistics, Boston University School of Public Health, Boston, MA USA; 5 Neurology Division, Department of Medicine, Boston University School of Medicine, Boston, MA USA; 6 Population Sciences Branch and Division of Intramural Research, National Heart, Lung, and Blood Institute of the National Institutes of Health, Bethesda, MA USA; 7 Mathematical and Statistical Computing Laboratory, Center for Information Technology, National Institutes of Health, Bethesda, MD USA; 8 Department of Mathematics and Statistics, Boston University, Boston, MA USA; 9 Hebrew SeniorLife, Boston, MA USA; 10 Harvard Medical School, Boston, MA USA; 11 Division of Cardiovascular Medicine, Department of Internal Medicine, University of Michigan, Ann Arbor, MI USA; 12 Cardiology Division, Department of Medicine, Beth Israel Deaconess Medical Center, Boston, MA USA; 13 55 Lake Avenue North, Worcester, MA 01655 USA

**Keywords:** Cardiovascular disease risk factors, Epidemiology, Circulation, mRNA, microRNA

## Abstract

**Background:**

Cardiometabolic (CM) risk factors are heritable and cluster in individuals. We hypothesized that CM risk factors are associated with multiple shared and unique mRNA and microRNA (miRNA) signatures. We examined associations of mRNA and miRNA levels with 6 CM traits: body mass index, HDL-cholesterol and triglycerides, fasting glucose, and systolic and diastolic blood pressures through cross-sectional analysis of 2812 Framingham Heart Study who had whole blood collection for RNA isolation for mRNA and miRNA expression studies and who consented to genetic research. We excluded participants taking medication for hypertension, dyslipidemia, or diabetes. We measured mRNA (*n* = 17,318; using the Affymetrix GeneChip Human Exon 1.0 ST Array) and miRNA (*n* = 315; using qRT-PCR) expression in whole blood. We used linear regression for mRNA analyses and a combination of linear and logistic regression for miRNA analyses. We conducted miRNA-mRNA coexpression and gene ontology enrichment analyses to explore relations between pleiotropic miRNAs, mRNA expression, and CM trait clustering.

**Results:**

We identified hundreds of significant associations between mRNAs, miRNAs, and individual CM traits. Four mRNAs (*FAM13A, CSF2RB, HIST1H2AC, WNK1*) were associated with all 6 CM traits (FDR < 0.001) and four miRNAs (miR-197-3p, miR-328, miR-505-5p, miR-145-5p) were associated with four CM traits (FDR < 0.05). Twelve mRNAs, including *WNK1*, that were coexpressed with the four most pleiotropic miRNAs, were also miRNA targets. mRNAs coexpressed with pleiotropic miRNAs were enriched for RNA metabolism (miR-505-5p), ubiquitin-dependent protein catabolism (miR-197-3p, miR-328) and chromatin assembly (miR-328).

**Conclusions:**

We identified mRNA and miRNA signatures of individual CM traits and their clustering. Implicated transcripts may play causal roles in CM risk or be downstream consequences of CM risk factors on the transcriptome. Studies are needed to establish whether or not pleiotropic circulating transcripts illuminate causal pathways for CM risk.

**Electronic supplementary material:**

The online version of this article (doi:10.1186/s12864-017-3533-9) contains supplementary material, which is available to authorized users.

## Background

Metabolic risk factors cluster in individuals and their presence is associated with increased risk for type II diabetes mellitus (T2DM) and cardiovascular disease (CVD) [1, 2]. Genome-wide association studies (GWAS) have identified hundreds of loci associated with cardiometabolic (CM) risk factors including body mass index (BMI), lipid levels, glucose levels, T2DM, and blood pressure [1–5]. In more recent years, circulating mRNA and miRNA transcriptional patterns have been linked to CVD and CM phenotypes [6–9].

Despite having identified molecular associations with CM risk factors at the genetic, mRNA, and miRNA levels, multidimensional interrelations of these molecular elements and how they interact to influence susceptibility to CM risk factors and CVD risk remain unknown. Recent studies have shown that circulating mRNA and miRNA transcript levels are heritable quantitative traits that are partly under genetic control [10]. The molecular underpinnings of complex CM diseases may be explained in part by genetic variation, mRNA and miRNA expression, and by miRNA–mRNA interaction [11]. Therefore, integrative analyses that incorporate multidimensional genomic data are necessary to investigate and characterize complex changes in the regulatory machinery and their effects on biological functions and complex CM phenotypes [6, 7].

With the goal of generating new insights into potential gene regulatory factors responsible for the clustering of CM risk factors, we examined the relations of circulating mRNA and miRNA expression levels to six CM traits: BMI, plasma lipid levels (HDL cholesterol [HDL-C], triglycerides [TG]), fasting glucose levels, and systolic (SBP) and diastolic (DBP) blood pressure. A high-throughput, unbiased approach was used to detect novel relationships among mRNAs and miRNAs across multiple CM traits since clustering of these traits is frequently seen clinically.

## Methods

### Study sample

The Framingham Heart Study (FHS) is a prospective, community-based observational study of CVD and its risk factors. We included participants from the FHS Offspring and Third Generation cohorts [12, 13]. We focused on 725 Offspring cohort participants (examination 8, 2005–2008) and 2087 Third Generation cohort participants (examination 2, 2008–2011) who had whole blood collection for RNA isolation for mRNA and miRNA expression studies and who consented to genetic research. We excluded participants taking medication for hypertension, dyslipidemia, or diabetes. Venous blood samples were obtained after an overnight fast and samples were stored using methods that maintain RNA stability (http://www.preanalytix.com/products/blood/RNA/paxgene-blood-rna-tube) [14]. All participants gave informed consent. The Boston University Medical Center Institutional Review Board approved FHS examination protocols and University of Massachusetts Medical School Review Board approved the miRNA and RNA profiling protocols.

### Risk factor definitions

FHS participants had a physician-administered history and physical examination including anthropometric measurements and a laboratory evaluation focused on CVD and its risk factors. Blood pressure was measured twice by a physician with the participant seated; the average of both measurements was used to calculate SBP and DBP. BMI was calculated by dividing the weight in kilograms by the square of height in meters (kg/m^2^). Fasting plasma glucose was measured using a hexokinase reagent kit (A-gent glucose test, Abbott Laboratories, Inc., South Pasadena, CA); the intra-assay CV was <3% [15]. Venous blood samples were collected in 0.1% EDTA tubes and plasma was separated by centrifugation. Plasma lipids levels were measured before freezing. Triglycerides were measured using an automated enzymatic assay. HDL-C was measured after dextran sulfate magnesium precipitation [16].

### mRNA expression profiling

Whole blood was collected in PAXgene (QIAGEN, Valencia, CA) tubes from each study participant after an overnight fast and stored at −80°C. RNA was extracted from whole blood using the PAXgene Blood RNA System Kit according to published methods [14]. RNA quality was validated using an Agilent 2100 Bioanalyzer (Agilent Technologies, Palo Alto, CA); A NanoDrop ND-1000 spectrophotometer was used to quantify RNA concentration (NanoDrop Technologies, Wilmington, DE). NuGEN’s WT-Ovation Pico RNA Amplification System was used to amplify 50 ng of total RNA, which was then labeled according to established protocols with FL-Ovation cDNA Biotin Module V2 (NuGEN, San Carlos, CA) [11].

### miRNA expression profiling

The same RNA sample was used for miRNA isolation. The high throughput Gene Expression and Biomarker Core Laboratory at the University of Massachusetts Medical School profiled 346 miRNAs isolated from whole blood (RNA isolation was performed by Asuragen, Inc, Austin, TX) in 2445 FHS Offspring and 3245 Third Generation cohort participants using TaqMan chemistry-based assays (Additional file 1: Methods). The initial miRNA list encompassed all TaqMan miRNA assays (774) available at the start of the study. If a miRNA was not expressed in any of 550 randomly selected samples, this miRNA was not examined in the entire cohort. Using this method, we restricted miRNA quantification in the overall cohort to the aforementioned 346 miRNAs. Methods used for cDNA conversion, preamplification, and quantification are reported in the Additional file 1: Methods. Among 70 replicate samples, >95% of the data points had coefficients of variation <10% (mean ~4%). As described previously, miRNA expression was quantified using cycle threshold (Ct), where higher Ct values reflect lower miRNA expression [14]. We analyzed 315 miRNAs that were expressed in at least 100 people.

### Statistical analyses

We report descriptive statistics using counts and percentages for binary variables and means ± standard deviations (SD) for continuous variables. We modeled mRNAs and miRNAs as response variables versus each CM risk factor, adjusting for age and sex. mRNA expression was quantified by log-2 transformed expression intensities. These models also adjusted for RNA processing variables and differential cell counts (imputed). RNA processing variables included isolation batch, RNA quality, concentration, and 260/280 ratio (ratio of absorbance at 260 and 280nm using a spectrophotometer) [14]. Differential cell counts (white blood count, and percent lymphocytes, monocytes, eosinophils, basophils) were imputed from mRNA expression values via partial least square (PLS) prediction, with cross-validated prediction accuracy estimates ranging from 0.25–0.89. We conducted modeling in the full sample and in two random, equal subsets (discovery and validation, Additional file 1), which preserved intact pedigrees. For each metabolic risk factor, we applied false discovery rate (FDR) calculations in the whole sample, the discovery set, and the validation set.

### mRNA analyses

The robust multichip analysis (RMA) algorithm [17] was applied using Affymetrix Power Tools (APT) for generation of signal values to yield an initially normalized dataset using log-2 transformed expression intensities. For a detailed description on data quality control and normalization, please refer to *Joehanes et al*. [18] Further adjustment of this dataset by Affymetrix quality control parameters (*all_probeset_mean, all_probeset_stdev, neg_control_mean, neg_control_stdev, pos_control_mean, pos_control_stdev, all_probeset_rle_mean, all_probeset_mad_residual_mean, mm_mean*), the first principal component of the dataset (*PC1*), batch (*Batch_Lump*), and a factor accounting for the non-random layout of probesets on the array (*ProbesetGroupDiff*) yielded a final normalized dataset. A pedigree-based mixed-effects model implemented in the R package "*pedigreemm (version 1.0-4)*" was applied to this final normalized dataset for analysis of differential gene expression. Age, sex, measured or imputed blood cell counts (*RBC, WBC, PLT, LY_PER, MO_PER, EO_PER, BA_PER, Retic_Per*) were included in the model as covariates.

### miRNA analyses

We used pedigree-based linear mixed-effects models (R package “lmekin”) to analyze continuous miRNA values (i.e., when Ct < 27) and logistic regression models to analyze binary values (i.e., Ct < 27 versus Ct > = 27). This was necessary because expression of miRNAs was not universal, but varied from 1.5–99.9% among miRNAs. The observed Ct values generally did not have a truncated normal distribution, which precluded Tobit modeling [19]. Furthermore, imputation produced extreme bi-modal distributions and was not acceptable for response data in linear modeling. Therefore, we employed an adaptive approach. For a given miRNA, if at least 90% of participants expressed it, we used the linear-model *p* value; if <10% of participants expressed it, we used the logistic-model *p* value; if between 10% and 90% of participants expressed the miRNA, we combined results from the two models. Specifically, we added their *X* ^2^ statistics and we calculated the *p*-value from the distribution of a *X* ^2^ variate with two degrees of freedom.

### miRNA-mRNA coexpression analysis

The coexpression analysis was performed on FHS samples for which miRNA and mRNA data were both available (*N* = 5626). Linear mixed models (R package “lmekin”) were used to conduct pairwise coexpression analyses for all profiled mRNAs (dependent variable, *N* = 17,318) and 280 miRNAs (independent variable) expressed in >200 samples, with fixed effects including age, sex, technical covariates, imputed cell types, surrogate variables (SV), and a random effect to account for family structure. As described above, the mRNA expression was quantified by log-2 transformed expression intensities. miRNA expression used Ct values with higher values reflected lower expression levels of miRNAs. Adjustment was made for technical covariates (11 for mRNA expression and 4 for miRNA expression). Surrogate variables (SVs) were computed from the mRNA expression data using the R package “SVA,” and 51 SVs associated with at least 1 miRNA at Bonferroni corrected *P* < 1.7 × 10^−4^ (0.05/280) were included in the statistical model. We chose SVs that correlated with at least 1 miRNA to adjust for additional hidden effects in the mRNA expression measurements that might have affected miRNA-mRNA correlations. The Benjamini-Hochberg method [20] was used to compute the false discovery rate (FDR). The significant miRNA-mRNA coexpression pairs were selected using FDR < 0.05.

### miRNA target databases

*For the top four multiple-trait associated miRNAs, we used OmniSearch to search for computationally predicted and experimentally validated targets. OmniSearch is a semantics-based integration system to search miRNA targets. OmniSearch provides access to multiple prediction databases, including miRDB, TargetScan, and miRanda, and experimentally validated including miRTarBase. The predicted miRNA-mRNA target pairs from these databases were compared with the miRNA-mRNA coexpression pairs identified in the current study.* The predicted miRNA-mRNA target pairs from these databases were compared with the miRNA-mRNA coexpression pairs identified in the current study.

### Pathway and gene ontology enrichment analysis

Co-expressed mRNAs for each miRNA were combined as a set and classified using Gene Ontology (GO) databases to identify potentially relevant biological processes. Fisher’s exact test was used to calculate enrichment *p* values of the overlapped genes in comparison with the number of co-expressed mRNAs and the number of genes in each GO biological process terms. Because of many GO terms duplicated, we only used 825 unique GO biological process terms as suggested by MsigDB [21]. We used a Bonferroni adjusted statistical significance threshold of *p* < 0.05/825 = 6.0 × 10^−5^.

All statistical analyses were performed using SAS software version 9.2 (SAS Institute Inc., Cary, NC, USA) or R software version 3.1.1 (R Foundation for Statistical Computing, Vienna, Austria).

## Results

Demographic and CM risk factor characteristics of the 2812 participants are shown in Table 1. The study sample consisted of middle-aged (49 ± 12 years) participants, a slight majority of whom were women (59%).

**Table 1:** Framingham Heart Study Offspring and Generation 3 Study participant characteristics^a^

Variable	Total Sample (*n* = 2812)	Third Generation Cohort (*n* = 2087)	Offspring Cohort (*n* = 725)
Age, y	49.2 (12)	44.6 (8)	62.6 (9)
Female sex, n (%)	1672 (59)	1207 (58)	465 (64)
Body mass index, kg/m^2^	26.9 (5)	26.9 (5)	26.7 (5.0)
Current smoking, n (%)	337 (12)	264 (12.7)	73 (10)
Prevalent diabetes mellitus, n (%)	38 (1)	26 (1.3)	12 (2)
Systolic blood pressure, mm Hg	117 (15)	114 (14)	125 (17)
Diastolic blood pressure, mm Hg	74 (10)	74 (9)	75 (10)
Serum glucose, mg/dL	95 (13)	93 (12)	100 (14)
Total cholesterol, mg/dL	192 (34)	189 (33)	203 (33)
High-density lipoprotein, mg/dL	62 (18)	62 (18)	63 (19)
Triglycerides, mg/dL	104 (67)	104 (70)	103 (57)

Data are presented as means ± standard deviation or number (percentage). Values reported were measured at enrollment

^a^Indicates absence of treated hypertension, cholesterol, or diabetes at baseline

### Circulating mRNAs in association with metabolic traits

Each CM trait was associated with multiple mRNA gene transcripts at FDR <0.05. TG was associated with the greatest number of circulating mRNAs (*N* = 5049), followed by BMI (*N* = 4826), HDL-C (*N* = 1768), DBP (*N* = 1499), SBP (*N* = 1019), and glucose (*N* = 1014).

**Fig. 1:**
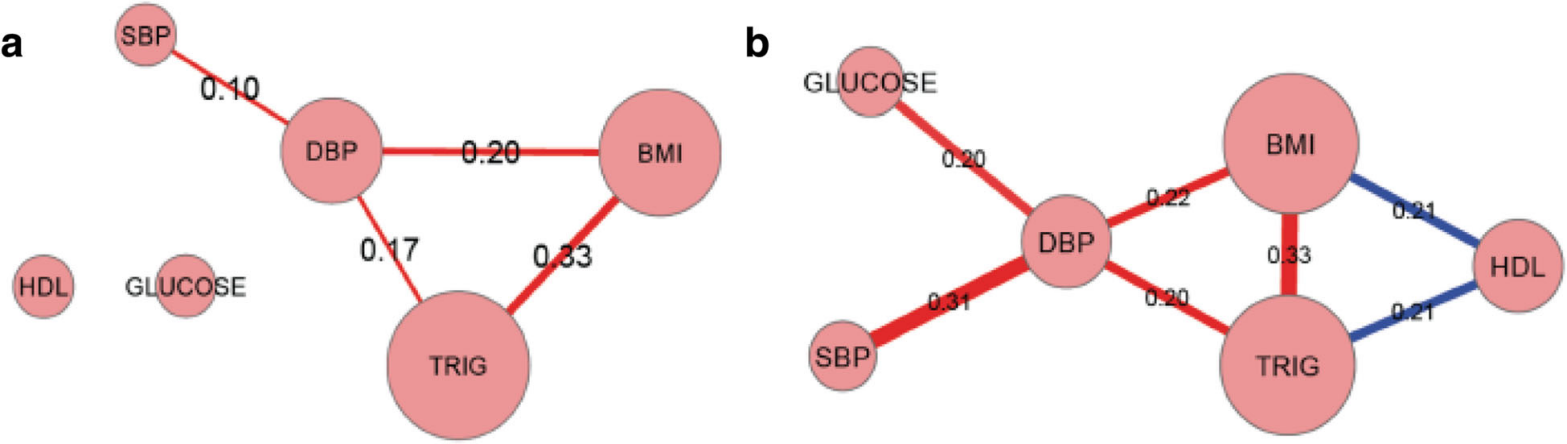
Similarity networks for whole blood miRNA (panel A) and mRNA (panel B) across cardiometabolic traits

Numerous transcripts were associated with multiple CM traits (Table 2 and Fig. 1). As shown in Fig. 1, genes associated with BMI shared associations with other CM traits, most notably DBP, TG, and HDL-C. Genes associated with both BMI and TG and those associated with BMI and DBP were positively correlated, whereas genes associated with both BMI and HDL-C were, in general, inversely correlated. SBP and DBP shared a large number of associated genes and the directionality of shared transcripts was concordant.

**Table 2:** Eighteen mRNAs^a^ with greatest pleiotropy across metabolic traits using a cut-off of FDR <0.001 to define significance of association

Gene Symbol	Gene Name	# Traits at FDR < 0.001	Traits Associated
*FAM13A*	family with sequence similarity 13, member A	6	All
*CSF2RB*	colony stimulating factor 2 receptor, beta, low-affinity	6	All
*HIST1H2AC*	histone cluster 1, H2ac	6	All
*WNK1*	WNK lysine deficient protein kinase 1	6	All
*ABCG1*	ATP-binding cassette, sub-family G (WHITE), member 1	5	All but glucose
*LSP1*	lymphocyte-specific protein 1	5	All but glucose
*LMBRD1*	LMBR1 domain containing 1	5	All but SBP
*ZNF721*	zinc finger protein 721	5	All but SBP
*PARP15*	poly (ADP-ribose) polymerase family, member 15	5	All but glucose
*ZNF644*	zinc finger protein 644	5	All but SBP
*AP2B1*	adaptor-related protein complex 2, beta 1 subunit	5	All but SBP
*PDS5B*	PDS5, regulator of cohesion maintenance, homolog B	5	All but glucose
*HIST1H4E*	histone cluster 1, H4e	5	All but HDL-C
*ZNF267*	zinc finger protein 267	5	All but HDL-C
*SAMHD1*	SAM domain and HD domain 1	5	All but HDL-C
*CAPN2*	calpain 2, (m/II) large subunit	5	All but HDL-C
*KLF10*	Kruppel-like factor 10	5	All but HDL-C
*CAST*	calpastatin	5	All but HDL-C

^a^Eighteen mRNAs represent all mRNAs associated with five or more cardiometabolic traits

Four circulating transcripts were associated with all six CM risk factors at an FDR <0.001 (Table 2), including several genes with known associations with cardiovascular and/or pulmonary disease: *FAM13A* (family with sequence similarity 13, member a) [22], *CSF2RB* (colony stimulating factor 2 receptor, beta) [23], *HIST1H2AC* (histone cluster 1, H2ac), and *WNK1* (WNK lysine deficient protein kinase 1) [4, 24]. An additional 14 transcripts were associated with five of the six CM traits (Table 2).

We conducted an analysis of all four of the most pleiotropic genes to examine relations to ‘energy metabolism’ or ‘cardiomyocyte function’ by searching these key words using the GeneRif database (https://www.ncbi.nlm.nih.gov/gene/about-generif), and found none of the four aforementioned mRNAs to have reported functions related to cardiomyocyte function or energy metabolism.

The top ten gene transcripts associated with each CM trait (BMI, HDL-C, TG, glucose, and SBP and DBP) are shown in Additional file 1: Tables S3–S8. The directionality and strength of association of the top four mRNAs are graphically depicted in Additional file 2: Figure S1B.

### Circulating miRNAs in association with cardiometabolic traits

Five CM traits were associated with multiple miRNAs (FDR < 0.05). TG was associated with the greatest number of miRNAs (*N* = 150), followed by DBP (*N* = 112), BMI (*N* = 99), SBP (*N* = 4). HDL-C and glucose were not associated with any circulating miRNAs at this FDR threshold.

Fifty miRNAs were associated with three or more CM traits and four were associated with four CM traits (miR-197-3p, miR-328, miR-505-5p, miR-145-5p) (Table 3 and Fig. 1). As shown in Fig. 1, miRNAs associated with BMI shared associations with other CM traits, most notably DBP and TG. miRNAs associated with both BMI and TG, and those associated with BMI and DBP, were positively correlated (reflected by line color, Fig. 1).

**Table 3:** Four miRNAs^a^ with greatest pleiotropy across metabolic traits using a cut-off of FDR <0.05 to define significance of association

Gene Symbol	Top Gene target^b^	# Traits at FDR < 0.05	Traits Associated
miR-505-5p	Major histocompatibility complex, class I (*MR1*)	4	All but HDL-C and glucose
miR-197-3p	SERTA domain containing 4 (*SERTAD4*)	4	All but HDL-C and glucose
miR-145-5p	ATP-binding cassette, subfamily E, member 1 (*ABCE1*)	4	All but HDL-C and glucose
miR-328	Transcription factor 7-like 2 (*TCF7L2*)	4	All but HDL-C and glucose

^a^Fifty miRNAs were associated with 3 traits (all 50 miRNAs were associated with the same 3 traits (HDL-C, DBP, and TG).^b^ Top target from miRDB (mirdb.org)

The top ten miRNAs associated with each CM trait (BMI, HDL-C, TG, glucose, and SBP and DBP) are shown in Additional file 1: Tables S9–S14. The directionality and strength of association of the top four miRNAs are graphically depicted in Additional file 2: Figure S1A.

### Replication results

Results of the separate analyses of discovery and validation sets revealed a high degree of concordance for mRNA results across all traits (Additional file 1: Table S1). Only two genes, *ARRDC3* and *CAPN2*, which were associated with glucose in the discovery set, failed to validate (at FDR <0.05) in our validation set; their validation set FDR values were 0.06. In contrast, owing to reduced power to detect miRNA-trait associations, far less concordance was noted between the discovery and replication sets for miRNA-trait associations (Additional file 1: Table S2).

### Coexpression analysis

**Fig. 2:**
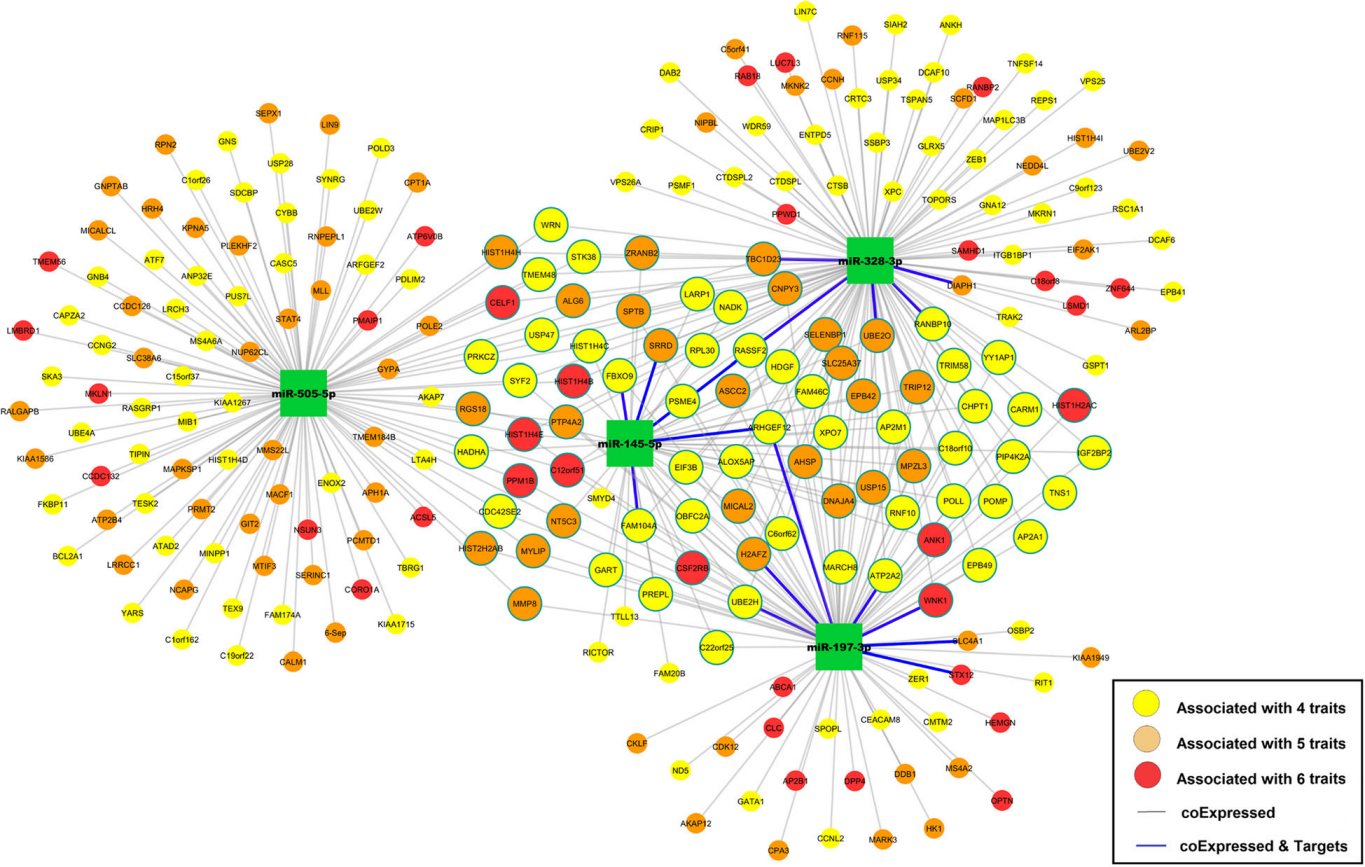
Coexpression network analysis

We identified coexpressed mRNAs for the four miRNAs that were associated with four CM traits (Table 3). These highly pleiotropic miRNAs were associated with a large number of mRNAs (1109 mRNAs in total; 396 coexpressed mRNAs for miR-505-5p, 241 for miR-197-3p, 177 for miR-145-5p, and 649 for miR-328). Notably, similar patterns of associations across traits were seen for mRNAs and miRNAs (Fig. 2).

**Fig. 3:**
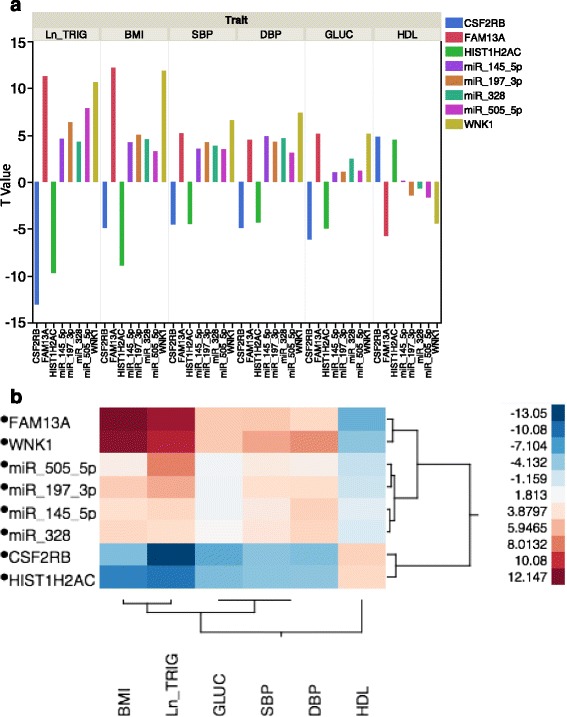
Pleotropic RNAs and miRNAs with heterogeneous effect directions across cardiometabolic traits. **a** Within trait associations showing heterogenous effect directions for pleiotropic mRNAs and miRNAs. **b** Heat map showing strength and directionality of associations between pleiotropic mRNA and miRNAs

Among the 1109 coexpressed mRNAs, 807 mRNAs were associated with at least one CM trait at FDR <0.1, and 249 were associated with more than three traits at FDR <0.1. A less restrictive FDR threshold was used in these analyses in light of the relatively low number of coexpressed mRNAs and miRNAs with CM trait associations included in this model. The coexpressed mRNAs for the four highly pleiotropic miRNAs were highly enriched for associations with CM traits (enrichment *P* < 1 × 10^−32^ by hypergeometric test). Figure 3 shows the miRNA-mRNA coexpression network for the four highly pleiotropic miRNAs and their coexpressed mRNAs (*n* = 249) that were each associated with at least four CM traits. Among the coexpressed mRNAs, 17 mRNAs were also miRNA targets reported by at least one miRNA target database, including *WNK1* for miR-197-3p.

Gene ontology enrichment analysis (Additional file 1: Table S15) revealed that the co-expressed mRNAs for miR-505-5p were enriched for genes involved in RNA metabolism (*P* = 3.5 × 10^−5^). The coexpressed mRNAs for miR-197-3p and miR-328 were enriched for cellular macromolecule catabolism (*P* = 7.67 × 10^−7^) and ubiquitin-dependent protein catabolism (*P* = 1.86 × 10^−10^). The coexpressed mRNAs for miR-328 were enriched for DNA packaging and chromatin assembly (*P* = 1.73 × 10^−5^). miRNAs coexpressed with miR-145 did not show significant enrichment for GO terms.

## Discussion

In a large, community-based cohort, we identified distinct as well as shared circulating transcriptomic signatures for CM risk factors. Four mRNAs were associated with all 6 CM risk factors (FDR <0.0001) and four miRNAs were associated with four CM risk factors (FDR <0.05). miRNAs associated with the greatest number of CM traits were coexpressed with many of the mRNAs that associated with multiple CM traits, including validated miR targets. Results of GO analyses revealed enrichment for processes relevant to regulation of gene expression and protein levels. These findings are consistent with causal roles of the implicated genes in CM risk. Alternatively, they may be due to downstream consequences of metabolic syndrome on the transcriptomic landscape. Functional studies are warranted to explore the mechanistic role of altered mRNA and miRNA expression in the pathogenesis of CVD and its CM risk factors.

To our knowledge, no prior study has performed a large-scale analysis of circulating miRNA and mRNA expression *across* CM traits. Several small studies, however, have examined mRNAs or miRNAs in relation to CM risk factors or disease. In the Young Finns Study [25], which included 71 participants, several circulating miRs were found to be associated with components of metabolic syndrome, including glucose and lipids. The authors also showed that down-regulated targets of two miRs, miR-1207-5p and miR-129-2-3p, were enriched in *PI3K* and *MAPK* pathways and that eight of 12 enriched pathways were downregulated in individuals with metabolic syndrome. Reflecting the translational relevance of our findings and validity of our approach, as discussed below, several of the most pleiotropic miRNAs and mRNAs identified in our analyses have been previously related to individual CM traits [26].

### Circulating mRNAs associated with multiple metabolic traits

Genes with the greatest pleiotropy across multiple CM traits (i.e. associated with all six CM traits at FDR <0.001) included *FAM13A* (family with sequence similarity 13, member A), *CSF2RB* (colony stimulating factor 2 receptor, beta), *HIST1H2AC* (histone cluster 1, H2ac), and *WNK1* (WNK lysine deficient protein kinase 1). *FAM13A* encodes a Rho GTPase activating protein involved in signal transduction. Variants in *FAM13A,* as well as *PARK2* and *RGS6,* have been associated with chronic lung disease in prior genome-wide association studies (GWAS) [27, 28]. In another recent GWAS involving over 180,000 participants, a SNP intronic to *FAM13A* was reported to be associated with HDL-C (*p* = 4 × 10^−12^) [22].

*CSF2RB* encodes a protein that is a common subunit to the three type I cytokine receptors (granulocyte-macrophage colony stimulating factor receptor, as well as the interleukin-3 and interleukin-5 receptors). As it has long been known that inflammatory cells, including macrophages, play important roles in the pathophysiology of atherogenesis, it is perhaps not surprising that recent animal work has demonstrated that *CSF2RB* expression affects monocyte and macrophage number and function in atherosclerotic lesions [23].

*HIST1H2AC* (histone cluster 1, H2ac) encodes Histone H2A type IC, one of the four core histones responsible for nucleosome structure in eukaryotic cells. Histones and other DNA-modifying/chromatin remodeling proteins play important roles as mediators of age-related DNA change and have been associated with cardiovascular risk factors, e.g., T2DM, and diseases, including atherosclerosis, myocardial infarction, and heart failure [29]. A GWAS involving 17,000 participants identified SNPs intronic to *HIS1H2AC* (including rs806971) that were associated with type 1 diabetes mellitus (*p* = 1.2 × 10^−10^) [30].

The WNK1 (WNK lysine deficient protein kinase 1) protein is a serine/threonine protein kinase that plays a role in angiogenesis associated with VEGF signaling. Overexpression of *WNK1* has also been linked to hypertension and hyperkalemia through alterations in sodium and potassium handling [4]. GWAS have linked two SNPs intronic to, or near, *WNK1* with TG levels as well as stroke risk [24].

Another gene exhibiting significant pleiotropy (5 traits; Table 2), ATP-binding cassette G1 (*ABCG1),* has been associated with total cholesterol levels in GWAS [24]. Lymphocyte-specific protein 1 (*LSP1*), which was associated with all metabolic traits except glucose (Table 2) harbors variants that are associated with both SBP and DBP [4].

### Circulating miRNAs associated with multiple cardiometabolic traits

miRNAs miR-505-5p, miR-197-3p, miR-145-5p, and miR-328 exhibited significant associations with BMI, SBP, DBP, and TG. miR-505-5p targets *SRSF1* (Serine/arginine-rich splicing factor 1). SRSF1, in turn, regulates endoglin, vascular endothelial growth factor A, and tissue factor, and controls a molecular senescence program in endothelial cells, leading to age-dependent vascular pathologies [31]. As shown in Table 3, the top gene target of miR-505 is major histocompatibility complex, class I (*MR1*). Recently, a novel susceptibility locus in *MR1* has been associated with coronary disease, likely as a result of dysregulated endothelial function and atherogenesis [31]. As with miR-505-5p, miR-145-5p is highly expressed in smooth muscle cells and controls smooth muscle cell differentiation and function, particularly in the context of metabolic syndrome [32]. Circulating miR-145 deletion results in impaired vascular contractility and differential expression of miR-145 in peripheral blood mononuclear cells has been shown to relate to hypertension [33]. Both miRNAs have strong signals that they are implicated in vascular function and susceptibility to cardiovascular disease.

In another recent study, circulating levels of miR-197 were associated with dyslipidemia in participants with metabolic syndrome and miR-197 levels correlation tightly to body mass index (*p* = 0.029) [34]. In contrast to the other pleiotropic miRNAs, miR-328 is highly expressed in platelets and has been associated with atrial fibrillation and cardiac hypertrophy, likely through *SERCA2A* dependent signaling pathways [35]. Notably, variants in the primary gene target of miR-328, transcription factor 7-like 2 gene (*TCF7L2*) [36], have been associated with T2DM in several genome wide association studies [37]. Prior associations and mRNA targets suggest that miRNAs 197 and 328 may play important roles in the regulation of gene networks influencing body mass and susceptibility to T2DM.

### miRNA-miRNA coexpression analyses and cardiometabolic traits

To better understand the molecular mechanisms underlying relations among circulating miRNAs, mRNAs, and CM traits, we conducted miRNA-miRNA coexpression analyses. Figure 3 displays the network of genes coexpressed with the most highly pleiotropic miRNAs. Seventeen mRNAs, including *WNK1*, that were coexpressed with the four most highly pleiotropic miRNAs, were each associated with multiple CM traits, and were found to be miR targets. We did not otherwise see extensive overlap between miRNA-mRNA targets and miRNA-mRNA coexpression. This may relate to the fact that miRNAs influence many non-traditional mRNA targets, and the effect of a miRNA on a single protein-coding gene target may be too small to be detected. Our findings do suggest, however, that key miRNAs (“hub” miRNAs, such as miR-197-3p) are shared across CM traits and co-express with genes previously associated with CM traits, e.g. *WNK1* [38]. We also identified notable associations between miR-145-5p with *ARHGEF12* (or *LARG*), a gene encoding Rho guanine nucleotide exchange factor 12, a vascular smooth muscle signaling protein that is required to develop salt-induced hypertension [39, 40]. Another notable finding was the association between miR-197-3p with *SLC4A1*, which encodes Band 3 anion transport protein. *SLC4A1* has been associated in GWAS with hypertension and its expression in the kidney is altered in animal models with altered sodium absorption [41, 42]. These findings suggest that miRNA-mRNA coexpression pairs may influence vascular phenotypes.

Gene ontology enrichment analysis (Additional file 1: Table S15) revealed that coexpressed mRNAs for the most pleiotropic miRNAs were enriched for RNA metabolism (miR-505-5p), ubiquitin-dependent protein catabolism (miR-197-3p and miR-328), and chromatin assembly (miR-328). The enriched GO terms (e.g., RNA metabolism, protein catabolism, and chromatin assembly) are all relevant to controlling gene expression and protein levels. For example, several genes involved in chromatin assembly (*HIST1H4E, HIST1H4B*) were highly co-expressed and are known to be related to several cardiovascular diseases [33].

Although our analyses revealed specific miRNAs and mRNAs associated and coexpressed with multiple CM traits, the global effect of these associations is likely to be complex. While “master regulation” may occur in specific settings, these data suggest that a cluster of gene expression changes is contributing to the many relevant pathways found in complex CM systems. It is well known that an individual miRNA can target multiple genes and each protein-coding gene can be regulated by several miRNAs but this complexity is compounded by the fact that most existing studies are performed with single miRNAs, limiting the interpretation of intricate observations. However, the unbiased approach of this study is a strength as it presents the complex findings as potential starting points for future mechanistic investigation.

*Study Limitations* Transcriptomic signatures may vary by cell type and patterns of mRNA and miRNA expression are known to differ between cell types. Since CM risk factors may influence white cell lineage differentiation, miRNA and mRNA levels in adults with CM traits may reflect differential leukocyte development. Nevertheless, in contrast to many prior analyses focusing on the circulating transcriptome, all observed associations were adjusted for white blood cell counts. Utilization of whole blood derived RNA for the analyses in our study does not provide detailed information on the specific cellular RNA source. We have previously observed that plasma derived extracellular miRNA and blood miRNA levels are often divergent, suggesting that distinct biological sources of RNA may reflect different biological processes and disease associations [43]. This conclusion is strengthened by our previous observations of both concordance and divergence amongst different blood sources of miRNA [44].

We excluded participants receiving several medications, including statins. Although this may have introduced bias (e.g., less severe CM phenotypes), this bias would likely have biased our results toward the null and does not threaten the validity of our findings. Finally, the FHS participants are largely middle-age adults of European ancestry. Generalizability to other younger individuals or those from other racial groups is uncertain.

## Conclusions

We found multiple circulating mRNAs and miRNAs that were associated with individual CM phenotypes and with their clustering. Our work supports the hypothesis that circulating transcriptomic patterns can be identified for CM traits and can be used to identify pathways involved in development and progression of CVD and its risk factors.

## Additional files

Additional file 1: Supplemental data. (DOCX 179 kb)
Additional file 2: Supplemental figures. (ZIP 249 kb)

## Abbreviations

CM: Cardiometabolic
CV: coefficient of variation
CVD: cardiovascular disease
DBP: diastolic blood pressure
GWAS: genome-wide association studies
HDL-C: HDL cholesterol
miRNA: microRNA
mRNA: messenger RNA
SBP: systolic
T2DM: type II diabetes mellitus
TG: triglycerides
