# Supplementary material for: Messenger RNA and MicroRNA transcriptomic signatures of cardiometabolic risk factors
Source: BMC Genomics. 2017 Feb 8;18:139. doi: 10.1186/s12864-017-3533-9 (PMC5299677; doi:10.1186/s12864-017-3533-9)
Supplement: Additional file 1: — Supplemental data. (DOCX 179 kb) [file 12864_2017_3533_MOESM1_ESM.docx]

**Supplemental Data (Tables 1-16, Supplementary Methods).**

| **BMI** | | | **HDL-C** | | | **Triglycerides** | | | **Glucose** | | | **Systolic Blood Pressure** | | | **Diastolic Blood Pressure** | | |
| --- | --- | --- | --- | --- | --- | --- | --- | --- | --- | --- | --- | --- | --- | --- | --- | --- | --- |
| **Top 10** | **FDR Disc** | **FDR**  **Val** | **Top 10** | **FDR Disc** | **FDR**  **Val** | **Top 10** | **FDR Disc** | **FDR**  **Val** | **Top 10** | **FDR Disc** | **FDR**  **Val** | **Top 10** | **FDR Disc** | **FDR**  **Val** | **Top 10** | **FDR Disc** | **FDR**  **Val** |
| *CX3CR1* | 1.8x10^-11^ | 3.6x10^-16^ | *CPA3* | 1.3x10^-10^ | 4.0x10^-15^ | *CPA3* | 3.8x10^-68^ | 1.3x10^-81^ | *KLF10* | 3.9x10^-4^ | 7.5x10^-9^ | *TSPAN2* | 4.3x10^-19^ | 3.8x10^-9^ | *TSPAN2* | 1.5x10^-11^ | 3.4x10^-4^ |
| *FAM13A* | 1.8x10^-11^ | 3.9x10^-16^ | *HDC* | 9.5x10^-8^ | 9.0x10^-18^ | *MS4A2* | 1.8x10^-67^ | 1.5x10^-64^ | *CPT1A* | 2.6x10^-6^ | 1.5x10^-6^ | *ANXA1* | 3.5x10^-9^ | 6.0x10^-7^ | *MS4A14* | 2.1x10^-8^ | 8.1x10^-5^ |
| *WNK1* | 3.0x10^-15^ | 3.3x10^-11^ | *MS4A2* | 6.4x10^-10^ | 2.7x10^-13^ | *HDC* | 5.0x10^-55^ | 1.2x10^-61^ | *PMAIP1* | 7.8x10^-4^ | 2.1x10^-4^ | *MS4A14* | 1.0x10^-11^ | 3.2x10^-4^ | *S100A10* | 5.3x10^-7^ | 8.1x10^-5^ |
| *TMEM56* | 2.8x10^-10^ | 1.2x10^-15^ | *AKAP12* | 9.1x10^-10^ | 6.2x10^-10^ | *AKAP12* | 2.4x10^-49^ | 1.3x10^-61^ | *ARRDC3* | 3.7x10^-7^ | **6.4x10^-2^** | *TAGAP* | 6.8x10^-11^ | 4.6x10^-4^ | *HIPK1* | 3.6x10^-5^ | 5.0x10^-5^ |
| *EPB42* | 2.0x10^-12^ | 1.7x10^-11^ | *GATA2* | 9.2x10^-6^ | 9.6x10^-11^ | *GATA2* | 1.2x10^-46^ | 1.9x10^-51^ | *TMEM2* | 8.8x10^-4^ | 9.0x10^-4^ | *GZMB* | 4.1x10^-9^ | 7.4x10^-5^ | *VIM* | 9.8x10^-5^ | 5.0x10^-5^ |
| *GSTK1* | 4.6x10^-11^ | 5.1x10^-12^ | *ENPP3* | 7.0x10^-6^ | 1.0x10^-8^ | *HRH4* | 1.5x10^-37^ | 4.3x10^-46^ | *PDK4* | 1.2x10^-2^ | 1.2x10^-4^ | *HIPK1* | 4.9x10^-9^ | 1.1x10^-3^ | *SPTB* | 2.0x10^-5^ | 8.1x10^-5^ |
| *DPP4* | 6.1x10^-10^ | 4.8x10^-13^ | *ABCG1* | 1.2x10^-7^ | 1.1x10^-6^ | *ENPP3* | 2.0x10^-40^ | 1.9x10^-37^ | *VCAN* | 4.4x10^-3^ | 1.9x10^-3^ | *SLC31A2* | 8.8x10^-7^ | 2.4x10^-5^ | *ABCA1* | 1.2x10^-2^ | 1.9x10^-7^ |
| *ALAS2* | 1.8x10^-10^ | 8.8x10^-12^ | *SCCPDH* | 1.7x10^-7^ | 8.1x10^-6^ | *ABCA1* | 8.7x10^-22^ | 6.0x10^-36^ | *HAVCR2* | 1.8x10^-2^ | 2.9x10^-4^ | *RAB11FIP1* | 7.1x10^-8^ | 4.6x10^-4^ | *TAGAP* | 1.5x10^-5^ | 8.1x10^-4^ |
| *ALOX5AP* | 2.1x10^-10^ | 2.7x10^-12^ | *CA8* | 1.1x10^-6^ | 9.1x10^-6^ | *SCCPDH* | 3.0x10^-32^ | 1.7x10^-24^ | *NLRC4* | 2.6x10^-2^ | 2.9x10^-4^ | *VIM* | 1.9x10^-7^ | 3.8x10^-4^ | *EPB42* | 5.3x10^-4^ | 4.0x10^-5^ |
| *NFKBIZ* | 1.3x10^-9^ | 2.6x10^-12^ | *HRH4* | 7.0x10^-6^ | 1.9x10^-6^ | *CA8* | 4.0x10^-27^ | 3.5x10^-20^ | *CAPN2* | 3.9x10^-4^ | **6.4x10^-2^** | *S100A10* | 1.9x10^-7^ | 4.6x10^-4^ | *RAB11FIP1* | 3.6x10^-5^ | 8.1x10^-4^ |

**Supplemental Table 1. FDR values for top 10 genes in the discovery and validation sets by CM trait**

**Supplemental Table 2. FDR q-Values for top 10 miRNAs in the discovery and validation Sets by CM trait**

| **BMI** | | | **HDL-C** | | | **Triglycerides** | | | **Glucose** | | | **Systolic Blood Pressure** | | | **Diastolic Blood Pressure** | | |
| --- | --- | --- | --- | --- | --- | --- | --- | --- | --- | --- | --- | --- | --- | --- | --- | --- | --- |
| **Top 10** | **FDR Disc** | **FDR**  **Val** | **Top 10** | **FDR Disc** | **FDR**  **Val** | **Top 10** | **FDR Disc** | **FDR**  **Val** | **Top 10** | **FDR Disc** | **FDR**  **Val** | **Top 10** | **FDR Disc** | **FDR**  **Val** | **Top 10** | **FDR Disc** | **FDR**  **Val** |
| miR-423-5p | **0.6339** | 1.07x10^-6^ | miR-29b-2-5p | **0.4519** | **0.3227** | miR-629-3p | 7.02X10^-5^ | 4.X10^-9^ | miR-497-5p | **0.9861** | **0.7533** | miR-197-3p | 0.0136 | **0.8379** | miR-671-3p | **0.9369** | **0.0999** |
| Let-7c | 0.0001 | 0.01546 | miR-339-5p | **0.9894** | **0.5889** | miR-25-5p | 0.0014 | 1.89X10^-10^ | miR-148a-3p | **0.3260** | **0.9529** | miR-328 | **0.1374** | **0.7615** | miR-145-5p | 0.0032 | **0.1387** |
| miR-320b | 0.0008 | 0.0331 | miR-629-5p | **0.9893** | **0.3923** | miR-505-5p | 0.0193 | 2.53X10^-6^ | miR-155-5p | **0.6842** | **0.7533** | miR-145-5p | **0.2231** | **0.8379** | miR-328 | 0.0092 | **0.1300** |
| miR-296-5p | 0.0297 | 0.0020 | miR-886-5p | **0.9894** | **0.5275** | miR-629-5p | **0.4913** | 1.32X10^-7^ | miR-320b | **0.4453** | **0.9529** | miR-505-5p | **0.2259** | **0.7615** | Let-7e-5p | 0.0253 | **0.0999** |
| miR-629-5p | **0.9181** | 0.0024 | miR-320b | **0.5564** | **0.4223** | miR-1180 | 0.0002 | 1.32X10^-7^ | miR-616-3p | **0.9861** | **0.7533** | Let-7g-3p | **0.3403** | **0.7615** | miR-296-5p | 0.0094 | **0.1333** |
| miR-29b_2-5p | 0.0116 | 0.0025 | miR-25-5p | **0.6959** | **0.3227** | miR-29b-2-5p | 0.0006 | 4.02X10^-6^ | miR-148b-3p | **0.6141** | **0.8802** | Let-7c | **0.2405** | **0.8379** | miR-301a-3p | 0.0491 | **0.0999** |
| miR-382-5p | **0.1551** | 0.0111 | miR-628-3p | **0.9281** | **0.4308** | miR-628-3p | 0.0001 | 3.10X10^-5^ | miR-296-5p | **0.5793** | **0.7662** | miR-629-3p | **0.2246** | **0.7615** | miR-197-3p | 0.0065 | **0.3549** |
| miR-342-3p | **0.4492** | 0.0112 | miR-1180 | **0.5247** | **0.5117** | miR-720 | **0.1520** | 4.02X10^-6^ | miR-328 | **0.6842** | **0.7533** | miR-671-3p | **0.7348** | **0.8805** | miR-365a-3p | **0.3624** | **0.1300** |
| miR-197-3p | 0.0100 | 0.0152 | miR-192-5p | **0.7625** | **0.4308** | miR-625-3p | 0.0040 | 1.82X10^-5^ | miR-342-3p | **0.8556** | **0.7533** | miR-329 | **0.2231** | **0.8379** | miR-433 | **0.0953** | **0.1387** |
| miR-126-3p | 0.0093 | 0.0168 | miR-29c-5p | **0.3256** | **0.8599** | miR-197-3p | 0.0003 | 9.86X10^-5^ | miR-375 | **0.6842** | **0.8457** | miR-296-5p | **0.2405** | **0.8379** | miR-629-5p | **0.5623** | **0.1188** |

**Supplemental Table 3. Top 10 genes in association with serum triglyceride levels (FDR for all top 10 genes p < 1.48x10^-48^, FDR q <0.05 highlighted)**

| **Gene Symbol** | **Gene Description** | **FDR**  **TG** | **FDR**  **BMI** | **FDR**  **HDL-C** | **FDR**  **DBP** | **FDR**  **SBP** | **FDR**  **Gluc** |
| --- | --- | --- | --- | --- | --- | --- | --- |
| *CPA3* | Carboxypeptidase A3 (mast cell) | 2.6x10^-152^ | 3.5x10^-14^ | 6.7x10^-28^ | 1.6x10^-4^ | 1.1x10^-1^ | 4.8x10^-3^ |
| *MS4A2* | Membrane-spanning 4-domains, subfamily A, member 2 (Fc fragment of IgE, high affinity I, receptor for; beta polypeptide) | 5.6x10^-134^ | 2.2x10^-8^ | 1.2x10^-25^ | 7.8x10^-5^ | 8.9x10^-2^ | 3.5x10^-2^ |
| *HDC* | Histidine decarboxylase | 1.6x10^-118^ | 3.2x10^-7^ | 4.7x10^-27^ | 2.6x10^-2^ | 8.1x10^-1^ | 7.4x10^-2^ |
| *AKAP12* | A kinase (PRKA) anchor protein 12 | 2.9x10^-111^ | 7.6x10^-6^ | 2.7x10^-22^ | 8.7x10^-5^ | 1.7x10^-1^ | 3.0x10^-2^ |
| *GATA2* | GATA binding protein 2 | 9.0x10^-99^ | 2.7x10^-12^ | 3.5x10^-18^ | 7.0x10^-5^ | 5.5x10^-2^ | 5.3x10^-3^ |
| *HRH4* | Histamine receptor H4 | 9.0x10^-86^ | 1.8x10^-4^ | 4.8x10^-14^ | 1.9x10^-2^ | 9.7x10^-2^ | 4.0x10^-2^ |
| *ENPP3* | Ectonucleotide pyrophosphatase/phosphodiesterase 3 | 2.6x10^-78^ | 3.1x10^-3^ | 2.1x10^-16^ | 1.4x10^-2^ | 6.9x10^-1^ | 2.1x10^-1^ |
| *ABCA1* | ATP-binding cassette, sub-family A (ABC1), member 1 | 4.0x10^-59^ | 2.5x10^-8^ | 1.2x10^-3^ | 5.9x10^-11^ | 1.4x10^-11^ | 9.6x10^-3^ |
| *SCCPDH* | Saccharopine dehydrogenase (putative) | 5.8x10^-57^ | 1.6x10^-10^ | 2.7x10^-15^ | 1.5x10^-3^ | 1.2x10^-1^ | 3.9x10^-1^ |
| *CA8* | Carbonic anhydrase VIII | 1.5x10^-48^ | 6.3x10^-8^ | 3.4x10^-14^ | 1.6x10^-1^ | 2.7x10^-1^ | 5.2x10^-1^ |

**Supplemental Table 4. Top 10 genes in association with Body Mass Index (FDR for all top 10 genes p < 2.0 x 10^-23^, FDR q <0.05 highlighted)**

| **Gene Symbol** | **Gene Description** | **FDR**  **TG** | **FDR**  **BMI** | **FDR HDL-C** | **FDR DBP** | **FDR SBP** | **FDR Gluc** |
| --- | --- | --- | --- | --- | --- | --- | --- |
| *FAM13A* | Family with sequence similarity 13, member A | 5.5x10^-26^ | 3.5x10^-33^ | 2.3x10^-6^ | 5.2x10^-4^ | 3.6x10^-5^ | 1.2x10^-4^ |
| *WNK1* | WNK lysine deficient protein kinase 1 | 4.5x10^-23^ | 9.6x10^-32^ | 5.5 x10^-4^ | 4.0x10^-10^ | 3.4x10^-8^ | 1.2x10^-4^ |
| *TMEM56* | Transmembrane protein 56 | 1.2x10^-29^ | 7.3x10^-31^ | 7.2x10^-6^ | 3.9x10^-3^ | 1.6x10^-2^ | 1.0x10^-4^ |
| *EPB42* | Erythrocyte membrane protein band 4.2 | 1.0x10^-30^ | 3.3x10^-29^ | 1.4x10^-4^ | 2.8x10^-10^ | 1.0x10^-6^ | 8.4x10^-2^ |
| *GSTK1* | Glutathione S-transferase kappa 1 | 2.2x10^-20^ | 3.2x10^-28^ | 2.4x10^-10^ | 4.4x10^-1^ | 6.9x10^-1^ | 1.7x10^-1^ |
| *DPP4* | Dipeptidyl-peptidase 4 | 3.4x10^-31^ | 1.3x10^-27^ | 1.2x10^-4^ | 4.2x10^-3^ | 4.0x10^-2^ | 9.2x10^-6^ |
| *ALAS2* | Aminolevulinate, delta-, synthase 2 | 7.0x10^-22^ | 5.9x10^-27^ | 1.3x10^-6^ | 4.1x10^-7^ | 3.1x10^-8^ | 2.5x10^-1^ |
| *ALOX5AP* | Arachidonate 5-lipoxygenase-activating protein | 1.2x10^-5^ | 6.7x10^-27^ | 6.6x10^-4^ | 5.8x10^-2^ | 3.0x10^-2^ | 6.6x10^-1^ |
| *NFKBIZ* | Nuclear factor of kappa light polypeptide gene enhancer in B-cells inhibitor, zeta | 1.5x10^-10^ | 1.1x10^-26^ | 6.3x10^-5^ | 3.4x10^-2^ | 3.0x10^-2^ | 4.3x10^-1^ |
| *CX3CR1* | Chemokine (C-X3-C motif) receptor 1 | 6.2x10^-14^ | 4.0x10^-22^ | 1.6x10^-2^ | 3.0x10^-7^ | 5.4x10^-6^ | 5.4x10^-2^ |

| **Gene Symbol** | **Gene Description** | **FDR**  **TG** | **FDR**  **BMI** | **FDR**  **HDL-C** | **FDR DBP** | **FDR**  **SBP** | **FDR Gluc** |
| --- | --- | --- | --- | --- | --- | --- | --- |
| *CPA3* | Carboxypeptidase A3 (mast cell) | 2.6x10^-152^ | 3.5x10^-14^ | 6.7x10^-28^ | 1.6x10^-4^ | 1.1x10^-1^ | 4.8x10^-3^ |
| *HDC* | Histidine decarboxylase | 1.6x10^-118^ | 3.2x10^-7^ | 4.7x10^-27^ | 2.6x10^-2^ | 8.1x10^-1^ | 7.4x10^-2^ |
| *MS4A2* | Membrane-spanning 4-domains, subfamily A, member 2 (Fc fragment of IgE, high affinity I, receptor for; beta polypeptide) | 5.6x10^-134^ | 2.2x10^-8^ | 1.2x10^-25^ | 7.8x10^-5^ | 8.9x10^-2^ | 3.5x10^-2^ |
| *AKAP12* | A kinase (PRKA) anchor protein 12 | 2.9x10^-111^ | 7.6x10^-6^ | 2.7x10^-22^ | 8.7x10^-5^ | 1.7x10^-1^ | 3.0x10^-2^ |
| *GATA2* | GATA binding protein 2 | 9.0x10^-99^ | 2.7x10^-12^ | 3.5x10^-18^ | 7.0x10^-5^ | 5.5x10^-2^ | 5.3x10^-3^ |
| *ENPP3* | Ectonucleotide pyrophosphatase/phosphodiesterase 3 | 2.6x10^-78^ | 3.1x10^-3^ | 2.1x10^-16^ | 1.4x10^-2^ | 6.9x10^-1^ | 2.1x10^-1^ |
| *ABCG1* | ATP-binding cassette, sub-family G (WHITE), member 1 | 1.1x10^-43^ | 1.3x10^-19^ | 2.6x10^-16^ | 1.2x10^-7^ | 3.7x10^-6^ | 5.8x10^-3^ |
| *SCCPDH* | Saccharopine dehydrogenase (putative) | 5.8x10^-57^ | 1.6x10^-10^ | 2.7x10^-15^ | 1.5x10^-3^ | 1.2x10^-1^ | 3.9x10^-1^ |
| *CA8* | Carbonic anhydrase VIII | 1.5x10^-48^ | 6.3x10^-8^ | 3.4x10^-14^ | 1.6x10^-1^ | 2.7x10^-1^ | 5.2x10^-1^ |
| *HRH4* | Histamine receptor H4 | 8.9x10^-86^ | 1.8x10^-4^ | 4.8x10^-14^ | 1.9x10^-2^ | 9.7x10^-2^ | 4.0x10^-2^ |

**Supplemental Table 5. Top 10 genes in association with serum high-density lipoprotein levels (FDR for all top 10 genes p <** **4.8x10^-14^, FDR q <0.05 highlighted)**

**Supplemental Table 6. Top 10 genes in association with diastolic blood pressure (FDR for all top 10 genes p < 3.4x10^-10^, FDR q <0.05 highlighted)**

| **Gene Symbol** | **Gene Description** | **FDR**  **TG** | **FDR BMI** | **FDR HDL-C** | **FDR DBP** | **FDR SBP** | **FDR Gluc** |
| --- | --- | --- | --- | --- | --- | --- | --- |
| *TSPAN2* | Tetraspanin 2 | 6.8x10^-1^ | 1.5x10^-4^ | 1.1x10^-2^ | 7.0x10^-17^ | 8.2x10^-31^ | 2.2x10^-1^ |
| *MS4A14* | Membrane-spanning 4-domains, subfamily A, member 14 | 2.0x10^-10^ | 7.6x10^-15^ | 5.7x10^-1^ | 1.8x10^-14^ | 3.9x10^-17^ | 5.6x10^-5^ |
| *S100A10* | S100 calcium binding protein A10 | 2.2x10^-7^ | 1.3x10^-13^ | 9.5x10^-1^ | 2.1x10^-13^ | 2.6x10^-13^ | 3.8x10^-4^ |
| *HIPK1* | Homeodomain interacting protein kinase 1 | 1.6x10^-10^ | 7.9x10^-15^ | 9.9x10^-1^ | 8.6x10^-12^ | 3.4x10^-14^ | 1.3x10^-6^ |
| *VIM* | Vimentin | 9.0x10^-12^ | 1.5x10^-19^ | 5.4x10^-2^ | 2.8x10^-11^ | 2.6x10^-13^ | 7.1x10^-6^ |
| *SPTB* | Spectrin, beta, erythrocytic | 3.7x10^-20^ | 4.0x10^-20^ | 4.8x10^-1^ | 3.7x10^-11^ | 5.5x10^-9^ | 6.0x10^-4^ |
| *ABCA1* | ATP-binding cassette, sub-family A (ABC1), member 1 | 4.0x10^-59^ | 2.5x10^-8^ | 1.2x10^-3^ | 5.9x10^-11^ | 1.4x10^-11^ | 9.6x10^-3^ |
| *TAGAP* | T-cell activation RhoGTPase activating protein | 1.7x10^-2^ | 4.4x10^-6^ | 5.0x10^-1^ | 1.3x10^-10^ | 7.8x10^-17^ | 2.3x10^-1^ |
| *EPB42* | Erythrocyte membrane protein band 4.2 | 1.0x10^-30^ | 1.2x10^-25^ | 1.4x10^-4^ | 2.8x10^-10^ | 1.0x10^-6^ | 8.4x10^-2^ |
| *RAB11FIP1* | RAB11 family interacting protein 1 (class I) | 6.0x10^-1^ | 3.7x10^-3^ | 7.8x10^-2^ | 3.4x10^-10^ | 6.8x10^-14^ | 5.3x10^-1^ |

| **Gene Symbol** | **Gene Description** | **FDR**  **TG** | **FDR**  **BMI** | **FDR HDL-C** | **FDR**  **DBP** | **FDR**  **SBP** | **FDR Gluc** |
| --- | --- | --- | --- | --- | --- | --- | --- |
| *TSPAN2* | Tetraspanin 2 | 6.8x10^-1^ | 1.5x10^-4^ | 1.1x10^-2^ | 6.9x10^-17^ | 8.2x10^-31^ | 2.2x10^-1^ |
| *ANXA1* | Annexin A1 | 6.6x10^-1^ | 2.1x10^-3^ | 2.4x10^-4^ | 1.2x10^-8^ | 9.3x10^-19^ | 6.3x10^-4^ |
| *MS4A14* | Membrane-spanning 4-domains, subfamily A, member 14 | 2.0x10^-10^ | 7.6x10^-15^ | 5.7x10^-1^ | 1.8x10^-14^ | 4.0x10^-17^ | 5.6x10^-5^ |
| *TAGAP* | T-cell activation RhoGTPase activating protein | 1.7x10^-2^ | 4.4x10^-6^ | 5.0x10^-1^ | 1.3x10^-10^ | 7.8x10^-17^ | 2.3x10^-1^ |
| *GZMB* | Granzyme B (granzyme 2, cytotoxic T-lymphocyte-associated serine esterase 1) | 8.0x10^-1^ | 1.2x10^-1^ | 1.4x10^-6^ | 2.5x10^-8^ | 7.3x10^-16^ | 2.1x10^-4^ |
| *HIPK1* | Homeodomain interacting protein kinase 1 | 1.6x10^-10^ | 7.9x10^-15^ | 9.9x10^-1^ | 8.7x10^-12^ | 3.4x10^-14^ | 1.3x10^-6^ |
| *SLC31A2* | Solute carrier family 31 (copper transporters), member 2 | 4.0x10^-1^ | 5.3x10^-7^ | 7.7x10^-1^ | 4.6x10^-8^ | 3.5x10^-14^ | 1.1x10^-1^ |
| *RAB11FIP1* | RAB11 family interacting protein 1 (class I) | 6.0x10^-1^ | 3.7x10^-3^ | 7.8x10^-2^ | 3.4x10^-10^ | 6.8x10^-14^ | 5.3x10^-1^ |
| *VIM* | Vimentin | 9.0x10^-12^ | 1.5x10^-19^ | 5.4x10^-2^ | 2.8x10^-11^ | 2.6x10^-13^ | 7.1x10^-6^ |
| *S100A10* | S100 calcium binding protein A10 | 2.2x10^-7^ | 1.3x10^-13^ | 9.5x10^-1^ | 2.1x10^-13^ | 2.6x10^-13^ | 3.8x10^-4^ |

**Supplemental Table 7. Top 10 genes in association with systolic blood pressure (FDR for all top 10 genes p < 2.6x10^-13^, FDR q <0.05 highlighted)**

**Supplemental Table 8. Top 10 genes in association with serum glucose levels (FDR for all top 10 genes p < 5.6x10^-07^, FDR q <0.05 highlighted)**

| **Gene Symbol** | **Gene Description** | **FDR**  **TG** | **FDR**  **BMI** | **FDR HDL-C** | **FDR DBP** | **FDR SBP** | **FDR Gluc** |
| --- | --- | --- | --- | --- | --- | --- | --- |
| *KLF10* | Kruppel-like factor 10 | 4.6x10^-21^ | 2.5x10^-8^ | 3.5x10^-2^ | 9.1x10^-7^ | 5.3x10^-10^ | 1.7x10^-15^ |
| *CPT1A* | carnitine palmitoyltransferase 1A (liver) | 5.8x10^-22^ | 3.3x10^-17^ | 5.0x10^-1^ | 2.2x10^-4^ | 7.0x10^-4^ | 1.7x10^-15^ |
| *PMAIP1* | phorbol-12-myristate-13-acetate-induced protein 1 | 1.7x10^-16^ | 2.3x10^-7^ | 2.0x10^-2^ | 2.4x10^-3^ | 2.0x10^-4^ | 6.7x10^-10^ |
| *ARRDC3* | arrestin domain containing 3 | 1.4x10^-26^ | 1.2x10^-16^ | 3.0x10^-11^ | 6.5x10^-3^ | 4.7x10^-2^ | 1.2x10^-09^ |
| *TMEM2* | transmembrane protein 2 | 7.2x10^-6^ | 2.5x10^-3^ | 2.5x10^-1^ | 1.4x10^-2^ | 6.6x10^-4^ | 1.2x10^-08^ |
| *PDK4* | pyruvate dehydrogenase kinase, isozyme 4 | 1.6x10^-6^ | 7.4x10^-9^ | 9.2x10^-1^ | 3.6x10^-4^ | 2.3x10^-6^ | 5.2x10^-08^ |
| *VCAN* | versican | 2.3x10^-8^ | 4.6x10^-8^ | 8.3x10^-1^ | 2.7x10^-2^ | 2.1x10^-3^ | 1.6x10^-07^ |
| *HAVCR2* | hepatitis A virus cellular receptor 2 | 7.9x10^-10^ | 2.5x10^-7^ | 7.3x10^-1^ | 2.6x10^-6^ | 2.5x10^-5^ | 2.7x10^-07^ |
| *NLRC4* | NLR family, CARD domain containing 4 | 4.0x10^-12^ | 1.4x10^-13^ | 4.5x10^-3^ | 2.2x10^-1^ | 1.8x10^-1^ | 4.7x10^-07^ |
| *CAPN2* | calpain 2, (m/II) large subunit | 2.8x10^-13^ | 2.5x10^-19^ | 2.2x10^-2^ | 1.0x10^-6^ | 1.2x10^-4^ | 5.6x10^-07^ |

**Supplemental Table 9. Top 10 miRNAs in association with serum triglyceride levels and strength of association (FDR) across other metabolic traits (FDR q <0.05 highlighted)**

| **miRNA** | **FDR**  **TG** | **FDR**  **BMI** | **FDR**  **HDL-C** | **FDR**  **DBP** | **FDR**  **SBP** | **FDR**  **Gluc** |
| --- | --- | --- | --- | --- | --- | --- |
| miR-629-3p | 2.6x10^-13^ | 3.7x10^-2^ | 4.3x10^-1^ | 1.1x10^-2^ | 1.2x10^-1^ | 8.2x10^-1^ |
| miR-629-5p | 3.9x10^-13^ | 6.7x10^-6^ | 6.6x10^-2^ | 6.2x10^-4^ | 1.7x10^-1^ | 5.8x10^-1^ |
| miR-25-5p | 3.9x10^-13^ | 1.4x10^-4^ | 1.5x10^-1^ | 3.4x10^-1^ | 4.9x10^-1^ | 6.3x10^-1^ |
| miR-505-5p | 3.9x10^-13^ | 7.0x10^-3^ | 4.3x10^-1^ | 1.3x10^-2^ | 4.1x10^-2^ | 7.2x10^-1^ |
| miR-1180 | 2.8x10^-12^ | 9.4x10^-3^ | 1.9x10^-1^ | 2.9x10^-3^ | 2.6x10^-1^ | 6.3x10^-1^ |
| miR-29b-2-5p | 5.4x10^-11^ | 8.0x10^-6^ | 6.6x10^-2^ | 6.9x10^-2^ | 3.0x10^-1^ | 6.3x10^-1^ |
| miR-628-3p | 5.4x10^-11^ | 4.1x10^-3^ | 1.5x10^-1^ | 3.7x10^-2^ | 3.2x10^-1^ | 10.0x10^-1^ |
| miR-720 | 7.8x10^-11^ | 1.4x10^-1^ | 3.0x10^-1^ | 2.1x10^-1^ | 8.4x10^-1^ | 5.8x10^-1^ |
| miR-625-3p | 4.4x10^-9^ | 9.0x10^-2^ | 2.7x10^-1^ | 1.1x10^-1^ | 3.6x10^-1^ | 8.2x10^-1^ |
| miR-197-3p | 7.9x10^-9^ | 2.0x10^-5^ | 5.0x10^-1^ | 6.2x10^-4^ | 8.1x10^-2^ | 7.7x10^-1^ |

**Supplemental Table 10. Top 10 miRNAs in association with body mass index and strength of association (FDR) across other metabolic traits (FDR q<0.05 highlighted)**

| **miRNA** | **FDR**  **TG** | **FDR**  **BMI** | **FDR**  **HDL-C** | **FDR**  **DBP** | **FDR**  **SBP** | **FDR**  **Gluc** |
| --- | --- | --- | --- | --- | --- | --- |
| miR-423-5p | 5.5x10^-8^ | 6.9x10^-9^ | 3.8x10^-1^ | 5.4x10^-3^ | 2.6x10^-1^ | 6.3x10^-1^ |
| Let-7c | 5.5x10^-8^ | 6.8x10^-7^ | 4.3x10^-1^ | 8.7x10^-2^ | 1.2x10^-1^ | 6.3x10^-1^ |
| miR-320b | 2.2x10^-4^ | 4.4x10^-6^ | 1.2x10^-1^ | 6.0x10^-1^ | 3.8x10^-1^ | 3.7x10^-1^ |
| miR-296-5p | 3.1x10^-8^ | 6.2x10^-6^ | 3.5x10^-1^ | 5.4x10^-4^ | 1.7x10^-1^ | 3.9x10^-1^ |
| miR-629-5p | 3.9x10^-3^ | 6.7x10^-6^ | 6.6x10^-2^ | 6.2x10^-4^ | 1.7x10^-1^ | 5.8x10^-1^ |
| miR-382-5p | 8.1x10^-3^ | 8.0x10^-6^ | 9.8x10^-1^ | 2.0x10^-3^ | 3.0x10^-1^ | 5.8x10^-1^ |
| miR-29b-2-5p | 5.4x10^-11^ | 8.0x10^-6^ | 6.6x10^-2^ | 6.9x10^-2^ | 3.0x10^-1^ | 6.3x10^-1^ |
| miR-342-3p | 2.2x10^-7^ | 8.7x10^-6^ | 4.3x10^-1^ | 2.0x10^-3^ | 2.8x10^-1^ | 3.9x10^-1^ |
| miR-197-3p | 7.9x10^-9^ | 2.0x10^-5^ | 5.0x10^-1^ | 6.2x10^-4^ | 8.1x10^-3^ | 7.7x10^-1^ |
| miR-126-3p | 9.8x10^-7^ | 2.2x10^-5^ | 4.3x10^-1^ | 2.2x10^-3^ | 1.8x10^-1^ | 7.0x10^-1^ |

**Supplemental Table 11. Top 10 miRNAs in association with high-density lipoprotein and strength of association (FDR) across other metabolic traits (FDR q <0.05 highlighted)**

| **miRNA** | **FDR**  **TG** | **FDR**  **BMI** | **FDR**  **HDL-C** | **FDR**  **DBP** | **FDR**  **SBP** | **FDR**  **Gluc** |
| --- | --- | --- | --- | --- | --- | --- |
| miR-629-5p | 3.9x10^-13^ | 6.7x10^-6^ | 6.6x10^-2^ | 6.2x10^-4^ | 1.7x10^-1^ | 5.8x10^-1^ |
| miR-886-5p | 4.5x10^-5^ | 3.4x10^-3^ | 6.6x10^-2^ | 1.7x10^-2^ | 5.4x10^-1^ | 8.2x10^-1^ |
| miR-339-5p | 4.8x10^-3^ | 2.1x10^-2^ | 6.6x10^-2^ | 3.1x10^-2^ | 3.0x10^-01^ | 5.8x10^-1^ |
| miR-29b-2-5p | 5.4x10^-11^ | 8.0x10^-6^ | 6.6x10^-2^ | 6.9x10^-2^ | 3.0x10^-01^ | 6.3x10^-1^ |
| miR-320b | 2.2x10^-4^ | 4.4x10^-6^ | 1.2x10^-1^ | 6.0x10^-1^ | 3.8x10^-01^ | 3.7x10^-1^ |
| miR-628-3p | 5.4x10^-11^ | 4.1x10^-3^ | 1.5x10^-1^ | 3.7x10^-2^ | 3.2x10^-01^ | 10.0x10^-1^ |
| miR-25-5p | 4.0x10^-13^ | 1.4x10^-4^ | 1.5x10^-1^ | 3.4x10^-1^ | 4.9x10^-01^ | 6.3x10^-1^ |
| miR-1180 | 2.8x10^-12^ | 9.4x10^-3^ | 1.9x10^-1^ | 2.9x10^-3^ | 2.6x10^-01^ | 6.3x10^-1^ |
| miR-192-5p | 6.1x10^-1^ | 1.5x10^-2^ | 2.3x10^-1^ | 6.2x10^-1^ | 7.3x10^-01^ | 7.2x10^-1^ |
| miR-29c-5p | 1.8x10^-1^ | 5.2x10^-1^ | 2.6x10^-1^ | 3.0x10^-1^ | 3.2x10^-01^ | 6.3x10^-1^ |

**Supplemental Table 12. Top 10 miRNAs in association with diastolic blood pressure and strength of association (FDR) across other metabolic traits (FDR q <0.05 highlighted)**

| **miRNA** | **FDR**  **TG** | **FDR**  **BMI** | **FDR**  **HDL-C** | **FDR**  **DBP** | **FDR**  **SBP** | **FDR**  **Gluc** |
| --- | --- | --- | --- | --- | --- | --- |
| miR-671-3p | 2.6x10^-6^ | 4.7x10^-4^ | 9.1x10^-1^ | 1.4x10^-4^ | 1.2x10^-1^ | 3.9x10^-1^ |
| miR-145-5p | 2.9x10^-5^ | 4.2x10^-4^ | 9.8x10^-1^ | 2.1x10^-4^ | 4.1x10^-2^ | 8.2x10^-1^ |
| miR-328 | 1.1x10^-4^ | 1.4x10^-4^ | 8.3x10^-1^ | 3.4x10^-4^ | 2.0x10^-2^ | 3.9x10^-1^ |
| miR-296-5p | 3.1x10^-8^ | 6.2x10^-6^ | 3.5x10^-1^ | 5.4x10^-4^ | 1.7x10^-1^ | 3.9x10^-1^ |
| miR-301a-3p | 6.0x10^-4^ | 1.4x10^-2^ | 9.6x10^-1^ | 5.4x10^-4^ | 1.9x10^-1^ | 10.0x10^-1^ |
| let-7e-5p | 8.6x10^-6^ | 3.6x10^-2^ | 4.3x10^-1^ | 5.4x10^-4^ | 1.9x10^-1^ | 9.4x10^-1^ |
| miR-197-3p | 7.9x10^-9^ | 2.0x10^-5^ | 5.0x10^-1^ | 6.2x10^-4^ | 8.1x10^-3^ | 7.7x10^-1^ |
| miR-629-5p | 3.9x10^-13^ | 6.7x10^-6^ | 6.6x10^-2^ | 6.2x10^-4^ | 1.7x10^-1^ | 5.8x10^-1^ |
| miR-93-5p | 2.0x10^-4^ | 1.9x10^-3^ | 4.6x10^-1^ | 6.2x10^-4^ | 1.9x10^-1^ | 9.8x10^-1^ |
| miR-365a-3p | 6.9x10^-4^ | 4.1x10^-2^ | 4.3x10^-1^ | 6.2x10^-4^ | 1.9x10^-1^ | 5.8x10^-1^ |

**Supplemental Table 13. Top 10 miRNAs in association with systolic blood pressure and strength of association (FDR) across other metabolic traits (FDR q <0.05 highlighted)**

| **miRNA** | **FDR**  **TG** | **FDR**  **BMI** | **FDR**  **HDL-C** | **FDR**  **DBP** | **FDR**  **SBP** | **FDR**  **Gluc** |
| --- | --- | --- | --- | --- | --- | --- |
| miR-197-3p | 7.9x10^-9^ | 2.0x10^-5^ | 5.0x10^-1^ | 6.2x10^-4^ | 8.1x10^-3^ | 7.7x10^-1^ |
| miR-328 | 1.1x10^-4^ | 1.4x10^-4^ | 8.3x10^-1^ | 3.1x10^-4^ | 2.0x10^-2^ | 3.9x10^-1^ |
| miR-145-5p | 2.9x10^-5^ | 4.2x10^-4^ | 9.8x10^-1^ | 2.1x10^-4^ | 4.1x10^-2^ | 8.2x10^-1^ |
| miR-505-5p | 3.9x10^-13^ | 7.0x10^-3^ | 4.3x10^-1^ | 1.3x10^-2^ | 4.1x10^-2^ | 7.2x10^-1^ |
| Let-7g-3p | 8.5x10^-5^ | 1.2x10^-2^ | 10.0x10^-1^ | 9.0x10^-3^ | 7.2x10^-2^ | 8.3x10^-1^ |
| Let-7c | 5.5x10^-8^ | 6.8x10^-7^ | 4.3x10^-1^ | 8.7x10^-2^ | 1.2x10^-1^ | 6.3x10^-1^ |
| miR-671-3p | 2.6x10^-6^ | 4.7x10^-4^ | 9.1x10^-1^ | 1.4x10^-4^ | 1.2x10^-1^ | 3.9x10^-1^ |
| miR-629-3p | 2.6x10^-13^ | 3.7x10^-2^ | 4.3x10^-1^ | 1.1x10^-2^ | 1.2x10^-1^ | 8.2x10^-1^ |
| miR-329 | 2.7x10^-1^ | 1.3x10^-1^ | 6.5x10^-1^ | 4.1x10^-2^ | 1.2x10^-1^ | 7.6x10^-1^ |
| miR-296-5p | 3.1x10^-8^ | 6.2x10^-6^ | 3.5x10^-1^ | 5.4x10^-4^ | 1.7x10^-1^ | 3.9x10^-1^ |

**Supplemental Table 14. Top 10 miRNAs in association with glucose and strength of association (FDR) across other metabolic traits (FDR q <0.05 highlighted)**

| **miRNA** | **FDR**  **TG** | **FDR**  **BMI** | **FDR**  **HDL-C** | **FDR**  **DBP** | **FDR**  **SBP** | **FDR**  **Gluc** |
| --- | --- | --- | --- | --- | --- | --- |
| miR-497-5p | 5.1x10^-1^ | 8.7x10^-1^ | 9.6x10^-1^ | 9.5x10^-1^ | 9.8x10^-1^ | 1.0x10^-2^ |
| miR-320b | 2.2x10^-4^ | 4.4x10^-6^ | 1.2x10^-1^ | 6.0x10^-1^ | 3.8x10^-1^ | 3.7x10^-1^ |
| miR-616-3p | 3.0x10^-8^ | 7.0x10^-3^ | 6.5x10^-1^ | 4.4x10^-2^ | 3.0x10^-1^ | 3.7x10^-1^ |
| miR-148a-3p | 9.5x10^-1^ | 9.4x10^-1^ | 8.0x10^-1^ | 1.1x10^-1^ | 6.6x10^-1^ | 3.7x10^-1^ |
| miR-155-5p | 1.9x10^-8^ | 4.5x10^-3^ | 9.0x10^-1^ | 2.9x10^-3^ | 1.9x10^-1^ | 3.7x10^-1^ |
| miR-296-5p | 3.1x10^-8^ | 6.2x10^-6^ | 3.5x10^-1^ | 5.4x10^-4^ | 1.7x10^-1^ | 3.9x10^-1^ |
| miR-342-3p | 2.2x10^-7^ | 8.7x10^-6^ | 4.3x10^-1^ | 2.0x10^-3^ | 2.8x10^-1^ | 3.9x10^-1^ |
| miR-375 | 1.8x10^-6^ | 6.1x10^-3^ | 4.6x10^-1^ | 4.2x10^-3^ | 2.9x10^-1^ | 3.9x10^-1^ |
| miR-148b-3p | 5.9x10^-1^ | 5.4x10^-1^ | 7.0x10^-1^ | 1.7x10^-1^ | 1.9x10^-1^ | 3.9x10^-1^ |
| miR-328 | 1.1x10^-4^ | 1.4x10^-4^ | 8.3x10^-1^ | 3.4x10^-4^ | 2.0x10^-2^ | 3.9x10^-1^ |

**Supplemental Table 15. Gene ontology enrichment analysis of mRNA coexpressed with top miRNAs***

| **miRNAs** | **Ontology category** | **# genes found in category** | **Overlapped genes** | **Fold- enrichment** | **P value** |
| --- | --- | --- | --- | --- | --- |
| miR-197-3p | cellular macromolecule catabolism | 440 | 21 | 3.48 | 5.82x10^-7^ |
|  | ubiquitin-dependent protein catabolism | 221 | 13 | 4.29 | 1.05x10^-5^ |
| miR-328 | ubiquitin-dependent protein catabolism | 221 | 30 | 3.86 | 1.86 x10^-10^ |
|  | cellular macromolecule catabolism | 440 | 37 | 2.39 | 7.67 x10^-7^ |
|  | DNA packaging | 359 | 31 | 2.45 | 3.63 x10^-6^ |
|  | chromatin assembly | 148 | 17 | 3.26 | 1.73 x10^-5^ |
| miR-505-5p | RNA metabolism | 477 | 24 | 2.49 | 3.51x10^-5^ |

*miR-145 did not show significant enrichment for gene ontology terms.

**Supplemental Table 16. Characteristics of participants in the Discovery and Validation Sets**

|  | **Discovery Set**  **N=2634** | **Validation Set**  **N=2587** |
| --- | --- | --- |
| Age | 54.9±13.3 | 55.3±13.2 |
| Male sex, n(%) | 1198 (45.5%) | 1178 (45.5%) |
| Current smoker, n(%) | 147 (5.6%) | 341 (13.2%) |
| Total cholesterol, mg/dL | 186.1 (36.5) | 187.1 (35.5) |
| HDL cholesterol, mg/dL | 58.9 (17.9) | 58.9 (17.9) |
| Triglyceride, mg/dL | 114.2 (72.5) | 115.6 (78.0) |
| Systolic blood pressure, mm Hg | 121.4 (16.9) | 121.9 (16.7) |
| Diastolic blood pressure, mm Hg | 73.8 (20.5) | 74.0 (9.8) |
| Glucose level, mg/dL | 100.9 (20.5) | 100.7 (21.3) |
| Body mass index, kg/m^2^ | 28.2 (5.7) | 28.1 (5.7) |
| Diabetes, n(%) | 248 (9.4%) | 225 (8.7%) |
| Coronary heart disease, n(%) | 123 (4.7%) | 127 (4.9%) |

**Legend. Data are presented as means ± standard deviation or number (percentage).**

**Values reported were measured at the baseline exam when RNA was collected.**

**Supplementary Methods**

Quantitative Real-Time Polymerase Chain Reaction (qRT-PCR) reactions were performed using a high-throughput technology (BioMark; Fluidigm, San Francisco, CA). TaqMan PCR Master Mix was mixed with Preamplified cDNA samples (No AmpErase UNG, Applied Biosystems, Foster City, CA) as well as Sample Loading Reagent (Fluidigm, San Francisco, CA). This mixture was pipetted into the inlets of the DynamicArray 96.96 chips (Fluidigm, San Francisco, CA). TaqMan miRNA Expression Assays (Applied Biosystems, Foster City, CA) were diluted 1:2 x with Assay Loading Reagent (Fluidigm, San Francisco, CA). Expression Assays and Loading Reagent were pipetted into the inlets of the DynamicArray 96.96. We placed the DynamicArray into the IFC Controller HX (Fluidigm, San Francisco, CA), which distributed the assays and samples into the reaction wells of the chip using microfluidic delivery. qRT-PCR reactions were performed as follows: 10 min at 95°C, 15 sec at 95°C, and lastly 1 min at 60°C for 30 cycles. Dynamic Arrays were in standard Society for Biomolecular Sciences plate format (384 well). There are no differences with respect to methods of pipetting between this format and other 384-well plate PCR preparations. The system is quantitative for miRNAs expressed at low levels. It can detect a single miRNA copy at 26-27 CT. Over 95% of the expression levels had CV <10% (mean ~4%).
