# Supplementary figures and images for: Messenger RNA and MicroRNA transcriptomic signatures of cardiometabolic risk factors
Source: BMC Genomics. 2017 Feb 8;18:139. doi: 10.1186/s12864-017-3533-9 (PMC5299677; doi:10.1186/s12864-017-3533-9)

A

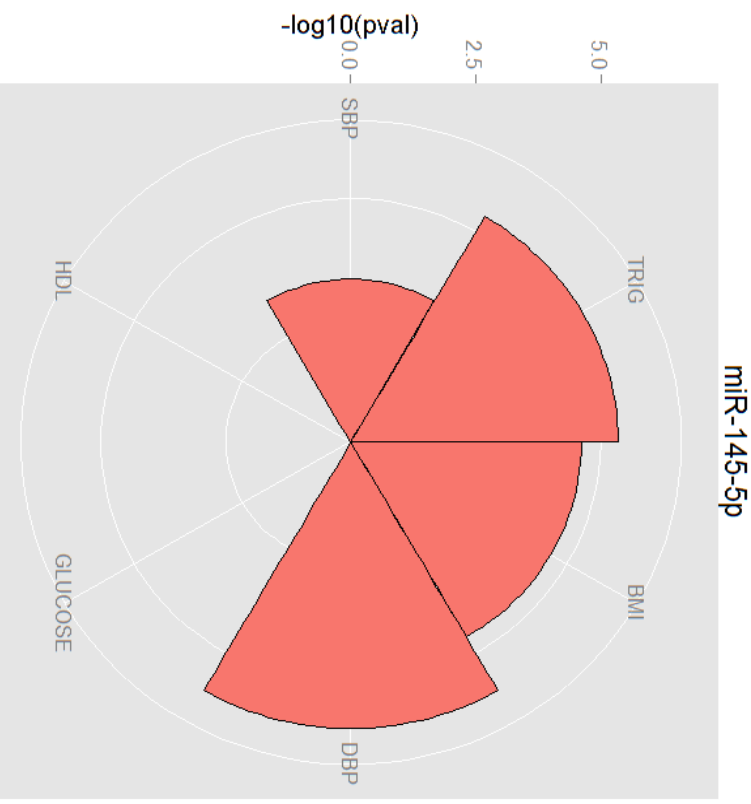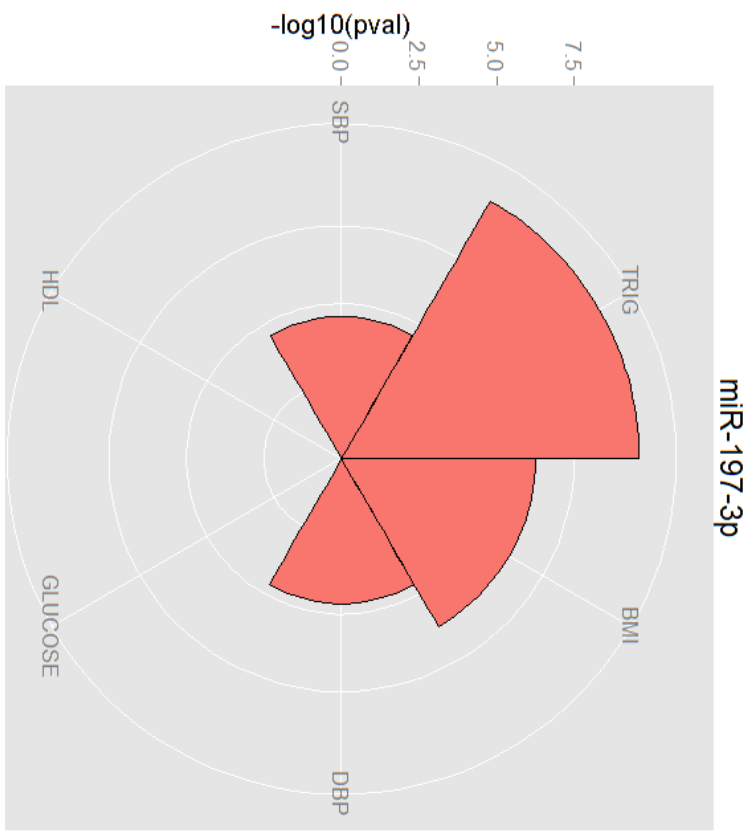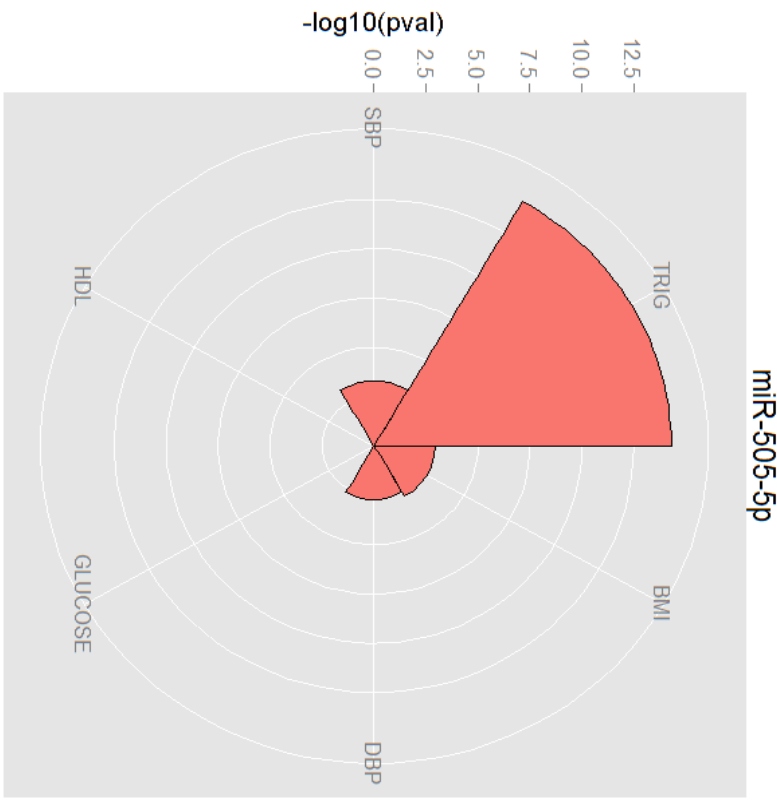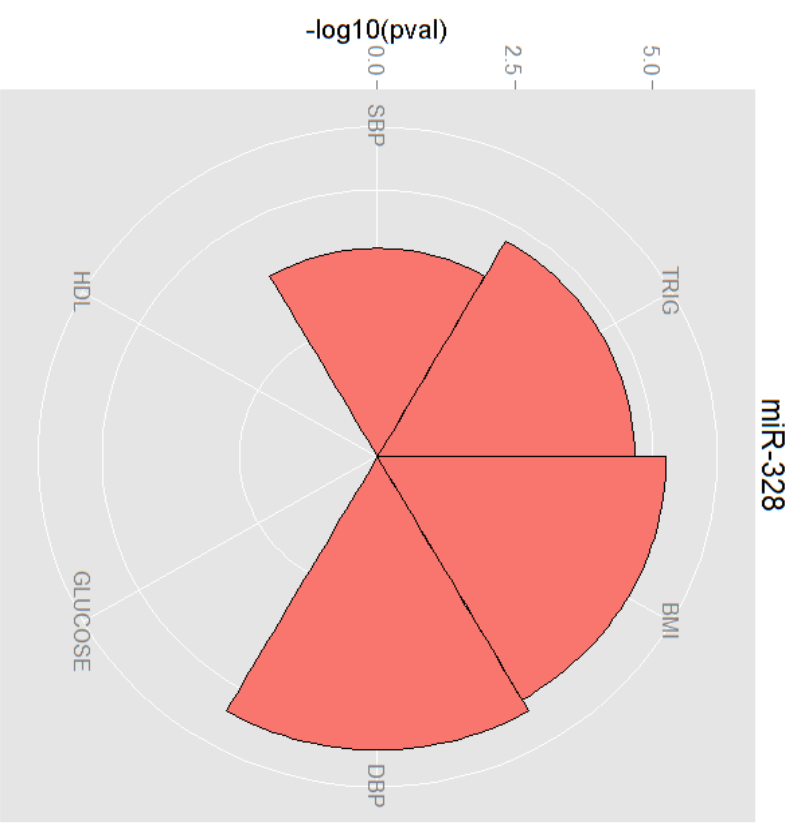

Supplement: Additional file 2: — Supplemental figures. (ZIP 249 kb) [file 12864_2017_3533_MOESM2_ESM.zip › SupplementalFig1A.pdf]

B

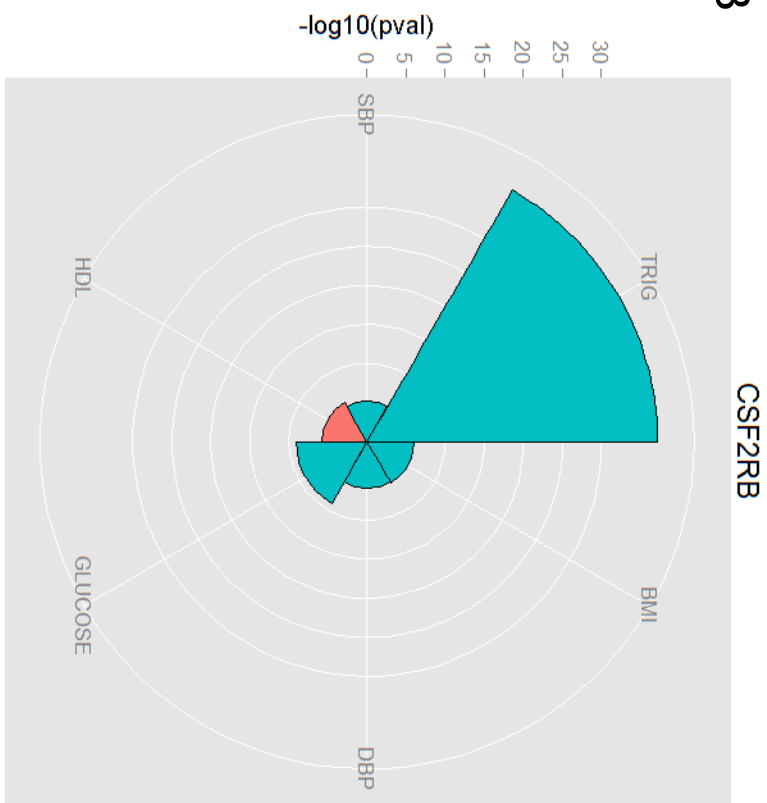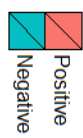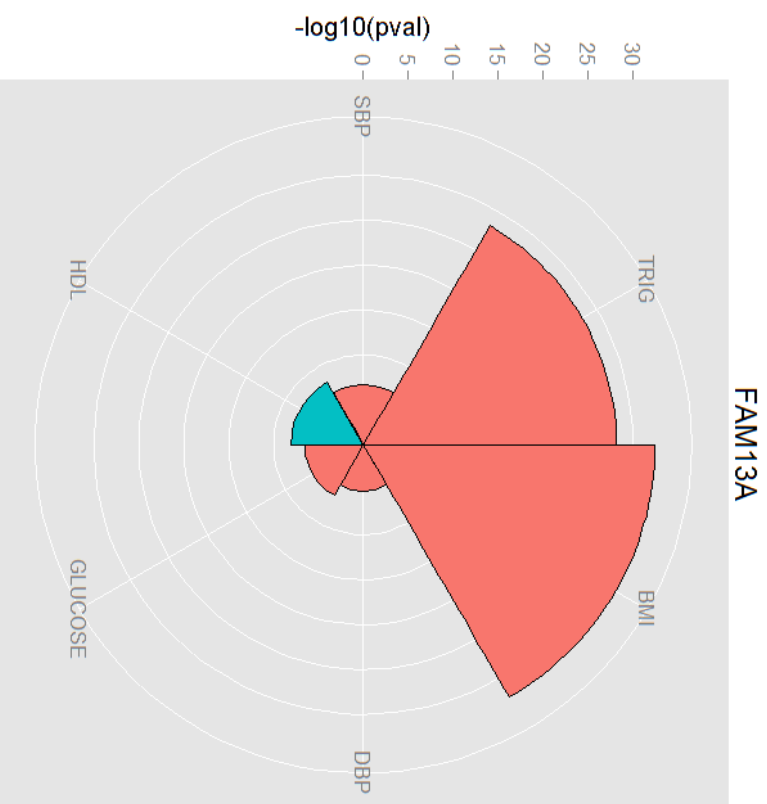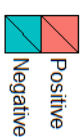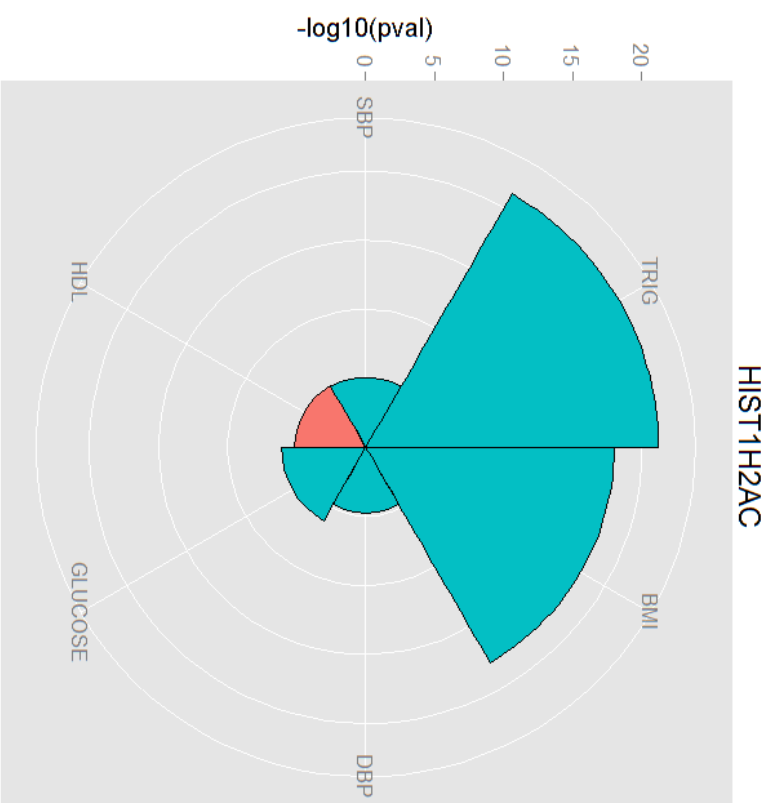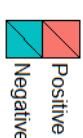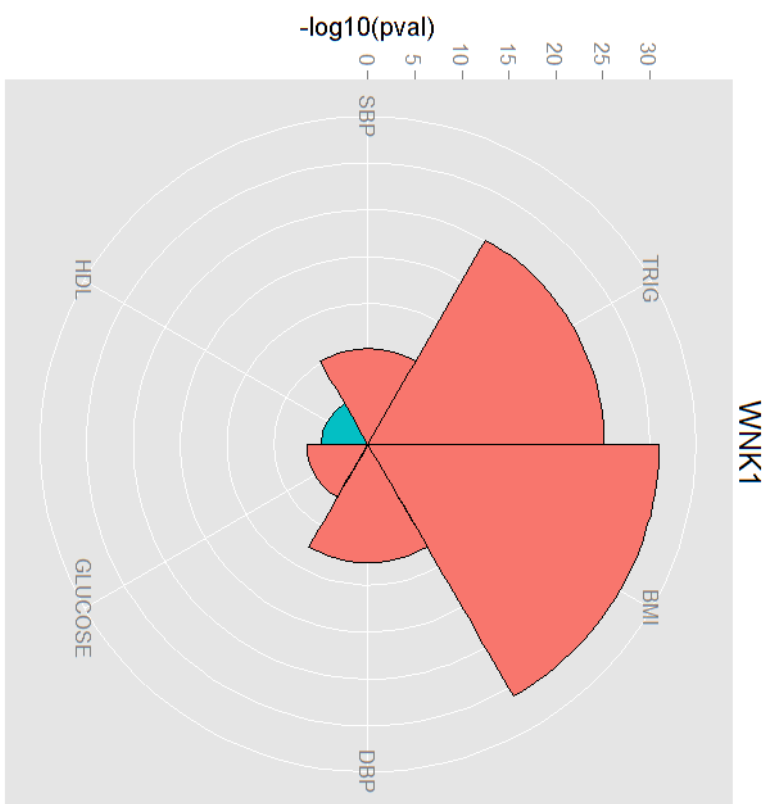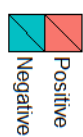

Supplement: Additional file 2: — Supplemental figures. (ZIP 249 kb) [file 12864_2017_3533_MOESM2_ESM.zip › SupplementalFig1B.pdf]
